# Improving Early Identification and Documentation of High-Risk Diabetic Foot Complications Through a Closed-Loop Clinical Audit at Almanagil Teaching Hospital

**DOI:** 10.7759/cureus.106403

**Published:** 2026-04-03

**Authors:** Elkhatab Abdulrahman Mustafa Abdulrahman, Mohamed Abdalla Elawad Wedatalla, Abdulrahman Abbas Yusuf Mohammed, Bidoor Alabbas, Zubaida kamal Abdalkhaliq Ahmed, Elsiddig Ameer Elsiddig Ahmed, Yusuf M. Y. Idris, Moneeb Elhaj Saad Eldaw, Hisham Osman Adam Suliman, Akram Mohamed Yagoub Elfaki, Mawia Ahmed, Mohammed Osman Ahmed Osman, Osama Mohamed Hamednalla Mohamed, Hadeel Fadlalla, Zeinab Abdelaziz Ali Ahmed, Osman Ali Osman Mustafa, Salma Abdullahi Hussein Abdi, Rania Boukhlet, Aisha Alzain, Mohamed Adel Abdalla Mohamed

**Affiliations:** 1 Emergency and Acute Medicine, Response Plus Medical Services LLC, Abu Dhabi, ARE; 2 Internal Medicine, Almanagil Teaching Hospital, Almanagil, SDN; 3 Internal Medicine, Port Sudan Teaching Hospital, Port Sudan, SDN; 4 Internal Medicine, University of Gezira, Wad Madani, SDN; 5 Emergency, Almanagil Teaching Hospital, Almanagil, SDN; 6 Surgery, Almanagil Teaching Hospital, Almanagil, SDN; 7 General Surgery, Almanagil Teaching Hospital, Almanagil, SDN; 8 Surgery, Dongola Specialised Hospital, Dongola, SDN; 9 Internal Medicine, The National Ribat University, Khartoum, SDN; 10 Internal Medicine, Ibri Regional Hospital, Ibri, OMN; 11 Internal Medicine, Burjeel Royal Hospital, Al Ain, ARE; 12 Surgery, Sudan Medical Specialization Board, Khartoum, SDN

**Keywords:** clinical audit, diabetic foot, diabetic foot infection, documentation quality, early detection, quality improvement, resource-limited settings, risk assessment

## Abstract

Background: Diabetic foot complications are a major cause of lower limb amputation, with delayed risk identification and inconsistent documentation contributing to poor outcomes, particularly in resource-limited settings. This clinical audit aimed to improve early identification and documentation of high-risk diabetic foot complications at Almanagil Teaching Hospital.

Methods: A closed-loop clinical audit was conducted over two cycles at Almanagil Teaching Hospital. Fifty consecutive medical records of patients with diabetes presenting with diabetic foot ulcers or suspected infection were reviewed in each cycle. Following a baseline assessment, a targeted quality improvement intervention was implemented, including staff education and the introduction of a standardized diabetic foot risk assessment and scoring tool. Documentation practices before and after the intervention were compared using descriptive and inferential statistics.

Results: At baseline, documentation was incomplete across most domains, with patient identifiers recorded in 22%-60% of records and key clinical assessments such as neuropathy, peripheral arterial disease, and infection documented in only 8%-18%. Following the intervention, documentation of patient and clinician identification reached 100%, while neuropathy and peripheral arterial disease assessments increased to 98% and 96%, respectively. Infection and skin integrity assessment improved from 8% to 94%, and objective risk stratification using total risk score calculation increased from 2% to 92%. Documentation of follow-up or clinical intervention rose from 22% at baseline to 96% post-intervention. All improvements between Cycle 1 and Cycle 2 were statistically significant (χ² tests, p < 0.001).

Conclusion: Implementation of a structured diabetic foot risk assessment tool supported by focused education was associated with marked improvements in documentation and early risk identification. These findings highlight the effectiveness of audit-driven quality improvement strategies in strengthening diabetic foot care processes and support their wider adoption in similar healthcare settings.

## Introduction

Diabetes mellitus is a chronic metabolic disorder with a rapidly rising global prevalence, driven by population aging, urbanization, and changes in lifestyle [1]. Among the numerous complications of diabetes, foot disorders -- particularly diabetic foot ulcers (DFUs) - remain a major cause of morbidity and healthcare burden worldwide [2]. Epidemiological data indicate that up to one-third of people with diabetes will develop a foot ulcer during their lifetime, and these ulcers are closely linked with significant rates of hospitalization, lower extremity amputation, and mortality [3]. DFUs not only diminish quality of life but also place substantial economic strain on patients and health systems, especially in regions with limited access to comprehensive diabetes care [4].

The pathogenesis of DFUs and subsequent infections is multifactorial, with peripheral neuropathy and peripheral arterial disease (PAD) as central contributory factors. Peripheral neuropathy leads to sensory loss, impairing the detection of minor trauma, while autonomic dysfunction contributes to skin dryness and fissure formation. PAD further exacerbates tissue ischemia and delays wound healing. These pathophysiological mechanisms interact to create an environment highly susceptible to ulceration and infection, which underscores the need for early recognition and systematic assessment of risk factors in patients with diabetes [5,6].

Diabetic foot infections (DFIs), ranging from superficial soft tissue involvement to deep infection and osteomyelitis, represent a critical turning point in the clinical course of DFUs. Infections are often the immediate precipitating cause of lower limb amputations, with studies showing that up to 15%-20% of moderately to severely infected DFUs eventually lead to amputation if not promptly and effectively managed. The clinical diagnosis of DFI is primarily based on local signs of inflammation or purulence, and severity classification systems such as the International Working Group on the Diabetic Foot (IWGDF) and Infectious Diseases Society of America (IDSA) frameworks are widely recommended to guide management decisions [7,8].

Guidelines from international expert bodies emphasize comprehensive evaluation and evidence-based treatment of DFIs. The IWGDF, in collaboration with IDSA, has updated its guidelines to incorporate the latest evidence on diagnosis, antimicrobial therapy, surgical intervention, and adjunctive care, highlighting that timely and appropriate management can reduce the risk of limb loss and improve outcomes. These recommendations stress the integration of vascular assessment, off-loading, glycemic control, and multidisciplinary care as essential components of effective foot infection management [8].

Despite established best-practice recommendations, substantial gaps in clinical practice persist, particularly in low- and middle-income countries and in resource-limited settings. Variations in the implementation of recommended screening protocols and inconsistency in risk stratification contribute to delayed diagnosis and suboptimal treatment. Research indicates that systematic approaches to foot assessment, risk scoring, and early intervention can significantly alter outcomes, yet many health systems lack structured quality improvement processes to ensure guideline adherence [9,10].

Clinical audit and quality improvement methodologies provide a pragmatic and structured approach to evaluating current clinical practice, identifying deficiencies, and implementing targeted interventions to improve patient care processes [5]. At Almanagil Teaching Hospital, preliminary observations and routine clinical practice reviews suggested inconsistencies in diabetic foot risk assessment, documentation, and early clinical decision-making, which may contribute to delayed recognition of high-risk DFUs and infections.

The primary objective of this clinical audit was to evaluate current practices in diabetic foot risk assessment and documentation at Almanagil Teaching Hospital against recognized evidence-based standards. Secondary objectives included assessing key components of peripheral neuropathy and PAD evaluation, infection identification, and risk stratification practices, and implementing targeted interventions to improve the completeness and accuracy of documentation.

## Materials and methods

This study was conducted as a closed-loop clinical audit and quality improvement project at Almanagil Teaching Hospital, Gezira State, Sudan, with the aim of evaluating and improving the early identification and documentation of high-risk diabetic septic foot complications. The project followed a structured Plan-Do-Study-Act (PDSA) methodology and was designed to assess current clinical practice, implement targeted interventions, and re-evaluate performance against predefined evidence-based standards.

The audit was conducted over a six-month period and consisted of two audit cycles separated by an intervention phase. The baseline audit (Cycle 1) was performed between 21 November and 31 December 2025, during which 50 consecutive medical records of adult patients with diabetes mellitus presenting with DFUs or suspected DFI were reviewed. Records were drawn from the internal medicine and surgical departments at Almanagil Teaching Hospital. Medical files lacking sufficient documentation for diabetic foot assessment or unrelated to diabetic foot presentations were excluded. Baseline data were collected to evaluate existing practices related to diabetic foot risk assessment, documentation, and early clinical management.

Data were collected using a locally developed structured audit checklist based on established international guidelines, particularly the recommendations of the IWGDF. The checklist was designed to capture key elements of diabetic foot risk assessment, clinical documentation, and initial management. To ensure content validity, the tool was reviewed by senior clinicians with experience in diabetic foot care. It was subsequently piloted on a small sample of medical records to assess clarity, feasibility, and completeness before full-scale implementation. Data collectors received standardized orientation on the use of the checklist to promote consistency in data extraction.

Following the analysis of Cycle 1 findings, a targeted quality improvement intervention phase was implemented between January and early February 2026. The intervention focused on addressing identified gaps and included structured educational sessions for medical staff on diabetic septic foot risk assessment, introduction of a standardized diabetic septic foot risk assessment and scoring tool, and reinforcement of multidisciplinary communication between internal medicine and surgical teams to support timely clinical decision-making and referral.

The re-audit (Cycle 2) was conducted between February and March 2026, during which 50 consecutive medical records of patients managed after implementation of the intervention were reviewed using the same audit tool and criteria applied in Cycle 1. This approach ensured consistency and allowed direct comparison between pre- and post-intervention performance.

Audit standards and assessment criteria were informed by internationally recognized guidelines, particularly those of the IWGDF, and were adapted to reflect local clinical practice and resource availability [8]. The audit evaluated documentation of key components of diabetic foot risk assessment, including assessment of peripheral neuropathy, evaluation of PAD, presence of foot deformities, history of previous ulceration or amputation, assessment of skin integrity and infection, assignment of risk level, calculation of total risk score, and documentation of follow-up or clinical intervention.

Data were collected using a structured, pre-designed audit checklist developed specifically for this project. Data from both audit cycles were entered into Google Forms (Google, Mountain View, California) and analyzed using both descriptive and inferential statistical methods. Descriptive statistics were used to summarize findings as frequencies and percentages. Inferential analysis was performed to assess differences in documentation and clinical practice between Cycle 1 and Cycle 2, with categorical variables compared using the chi-square test. A p-value of less than 0.05 was considered statistically significant. Absolute percentage change was also calculated to quantify the magnitude of improvement following the intervention.

Ethical approval for this quality improvement project was obtained from the Institutional Review Board of Almanagil Teaching Hospital under IRB Reference Number: IRB 17\MTH\1990. As the project involved review of routine clinical documentation with no direct patient contact and no alteration of patient management, individual informed consent was not required. All data were anonymized before analysis to ensure patient confidentiality and compliance with institutional governance and ethical requirements.

## Results

A total of 50 medical records were reviewed in each audit cycle. During Cycle 1, documentation of patient identification variables was incomplete, with patient name recorded in 27 records (54%), age in 21 records (42%), ID or file number in 11 records (22%), and date in 18 records (36%). Clinician-related documentation was similarly variable, with clinician name recorded in 30 records (60%) and department or consultant documented in 28 records (56%). Following the intervention, Cycle 2 demonstrated complete compliance for all patient and clinician identification fields, with documentation rates reaching 100% across all corresponding variables. These improvements were statistically significant (χ² ranging from 22.56 to 44.16, all p < 0.001).

Baseline documentation of key components of the clinical risk assessment was low in Cycle 1. Neuropathy assessment was recorded in 6 records (12%), PAD assessment in 8 records (16%), and foot deformity or skin condition assessment in 9 records (18%). Documentation of previous ulceration or amputation history was present in 7 records (14%), while infection and skin integrity assessment were documented in only 4 records (8%). In Cycle 2, substantial improvements were observed, with neuropathy assessment documented in 49 records (98%), PAD assessment in 48 records (96%), foot deformity and skin condition assessment in 49 records (98%), previous ulcer or amputation history in 49 records (98%), and infection and skin integrity assessment in 47 records (94%). All improvements were statistically significant (χ² values between 61.73 and 71.27, p < 0.001).

Footwear-related risk assessment and patient capacity variables also showed marked improvement between audit cycles. During Cycle 1, footwear risk assessment was documented in 3 records (6%), footwear risk factors in 2 records (4%), and patient self-care capacity in 2 records (4%). Following the intervention, documentation rates increased to 46 records (92%) for all three variables in Cycle 2, representing statistically significant improvements (χ² values ≥ 70.59, p < 0.001).

At baseline, patient-related modifiers were documented in 2 records (4%), and objective risk stratification using the total risk score calculation was recorded in only 1 record (2%). After implementation of the standardized assessment tool, documentation of patient-related modifiers increased to 46 records (92%), and total risk score calculation was recorded in 46 records (92%) during Cycle 2. These changes represented the largest absolute improvements observed in the audit and were statistically significant (χ² = 74.08 and 77.72, respectively; p < 0.001).

Documentation of follow-up planning or clinical intervention was present in 11 records (22%) during Cycle 1. This increased to 48 records (96%) in Cycle 2 following the intervention, indicating a substantial improvement in documented clinical action after risk identification. The difference in documentation rates between audit cycles was statistically significant (χ² = 53.58, p < 0.001), with higher rates observed in Cycle 2 (Table 1).

**Table 1:** Comparison of documentation completeness and clinical assessment practices between audit cycles at Almanagil Teaching Hospital (n = 50 per cycle) Data are presented as absolute frequencies followed by percentages in brackets, calculated using a denominator of 50 medical records for each audit cycle. Improvements represent absolute percentage-point changes between Cycle 1 (baseline) and Cycle 2 (post-intervention). Differences between audit cycles were assessed using the chi-square test for categorical variables (df = 1). A p-value < 0.05 was considered statistically significant.

Category	Cycle 1, n (%)	Cycle 2, n (%)	Improvement (Δ%)	χ² (df = 1)	p-value
Patient name	27 (54%)	50 (100%)	+46	27.33	<0.001
Age	21 (42%)	50 (100%)	+58	38.08	<0.001
ID/file number	11 (22%)	44 (88%)	+66	41.37	<0.001
Date	18 (36%)	50 (100%)	+64	44.16	<0.001
Clinician name	30 (60%)	50 (100%)	+40	22.56	<0.001
Department/consultant	28 (56%)	50 (100%)	+44	25.70	<0.001
Neuropathy assessment	6 (12%)	49 (98%)	+86	71.27	<0.001
Peripheral artery disease assessment	8 (16%)	48 (96%)	+80	61.73	<0.001
Foot deformity and skin condition assessment	9 (18%)	49 (98%)	+80	62.44	<0.001
Previous ulcer/amputation history	7 (14%)	49 (98%)	+84	68.22	<0.001
Infection and skin integrity assessment	4 (8%)	47 (94%)	+86	70.59	<0.001
Footwear risk assessment	3 (6%)	46 (92%)	+86	70.59	<0.001
Footwear risk factors	2 (4%)	46 (92%)	+88	74.08	<0.001
Patient self-care capacity	2 (4%)	46 (92%)	+88	74.08	<0.001
Patient-related modifiers	2 (4%)	46 (92%)	+88	74.08	<0.001
Risk level assignment (total score)	1 (2%)	46 (92%)	+90	77.72	<0.001
Follow up/interventions	11 (22%)	48 (96%)	+74	53.58	<0.001

Notably, the most clinically relevant improvements were observed in key domains of diabetic foot risk assessment. Documentation of neuropathy assessment increased markedly from 12% in Cycle 1 to 98% in Cycle 2, while PAD assessment improved from 16% to 96%. Similarly, infection and skin integrity assessment demonstrated a substantial increase from 8% to 94%. In addition, critical risk stratification practices showed pronounced enhancement, with the total risk score calculation increasing from 2% to 92%. These findings highlight significant improvements in the early identification and clinical evaluation of high-risk diabetic foot conditions following the intervention.

## Discussion

This clinical audit at Almanagil Teaching Hospital demonstrated significant improvement in the documentation and clinical assessment of diabetic foot risk factors and management following a targeted quality improvement intervention that combined structured assessment tools with focused clinical education. Baseline adherence to evidence-based components of diabetic foot assessment was low, mirroring findings from similar quality improvement projects that highlighted gaps in routine foot examinations and risk documentation in everyday clinical practice. Interventional studies have shown that standardized foot assessment strategies can dramatically increase compliance with recommended screening practices, improving early detection of peripheral neuropathy and PAD and ensuring high-risk patients are appropriately identified and managed [11,12].

The improvements observed in this audit are consistent with reports from other quality improvement initiatives that adopted structured diabetic foot screening protocols. In a primary care setting in India, the introduction of a standardized foot assessment policy combined with staff training and periodic audit raised compliance from 0% to over 80% within six months, illustrating the potential for systematic processes to overcome longstanding barriers to routine assessment [12]. Similarly, the use of clinical decision support and education in outpatient settings has been associated with increased rates of documented foot examinations, reinforcing the role of simple, targeted interventions in improving care delivery for patients with diabetes [13].

Infectious complications of DFUs are among the most serious outcomes of inadequate risk assessment. The IWGDF and IDSA clinical classification systems emphasize early clinical recognition and stratification of infection severity to guide timely management and prevent progression to osteomyelitis or limb loss [8]. The substantial increase in documented infection assessments following our intervention -- reflected in both higher frequencies and statistically significant differences -- supports the notion that structured clinical tools can enhance early recognition of DFI, which is critical for improving patient outcomes.

Peripheral neuropathy and PAD are well-established risk factors for DFUs and are strongly associated with increased risks of infection, hospitalization, and lower-limb amputation when not identified early through systematic screening [14]. Contemporary clinical practice guidelines emphasize the importance of early and routine screening for peripheral neuropathy and PAD in all patients with diabetes, as these conditions represent major predictors of diabetic foot ulceration and limb complications [15-17]. The dramatic improvements in documentation of neuropathy and PAD assessments in Cycle 2 suggest that targeted audit and feedback mechanisms can effectively embed these guideline recommendations into routine clinical workflows.

Enhanced documentation of previous ulceration or amputation history and total risk score calculation is important because prior history and objective risk stratification are powerful predictors of future ulceration and infection. Prospective studies have shown that a history of foot complications increases the risk of recurrent problems and amputation, underscoring the need for comprehensive documentation and stratification during each encounter [18]. The high levels of compliance achieved in these domains following the intervention indicate successful adoption of systematic risk assessment practices that align with best practice recommendations.

While this audit focused on documentation and process measures, evidence from the literature suggests that improved assessment processes can lead to better clinical outcomes. Quality improvement reports have linked standardized foot assessment and referral pathways with enhanced rates of specialist care engagement and earlier interventions, which are associated with lower rates of severe complications and amputations [12]. Although our study did not measure long-term clinical outcomes directly, the improvements in assessment and documentation are promising intermediate markers of enhanced quality of care.

Despite these positive findings, several limitations should be acknowledged. Firstly, this audit evaluated documentation practices rather than direct observation of clinical behavior and therefore may be subject to documentation bias, where recorded information may not fully reflect actual clinical practice. In addition, the retrospective nature of data collection introduces the possibility of observer bias during data extraction. Furthermore, this study evaluated the short-term impact of the intervention; therefore, the sustainability of improved practice over time remains uncertain without ongoing audit cycles and reinforcement strategies.

Finally, the audit did not explore the patient perspective, including experiences of foot care education, footwear counseling, or engagement with self-care practices. Evidence suggests that patient education is a key determinant of preventive foot care behaviors and can influence outcomes independently of clinical assessment practices [11]. Future quality improvement efforts should consider integrated strategies that include components of patient education and empowerment.

In summary, this clinical audit demonstrates that a targeted quality improvement intervention significantly improved the completeness of diabetic foot risk assessment and documentation among patients at Almanagil Teaching Hospital. These findings align with broader evidence indicating that structured assessment protocols and audit-feedback mechanisms can enhance adherence to clinical best practices. Even in resource-limited settings -- characterized by limited access to standardized assessment tools, structured documentation systems, and formal training in diabetic foot risk evaluation -- this audit demonstrated that simple, low-cost interventions such as structured assessment forms, staff education, and visual aids can substantially improve clinical documentation and risk identification practices. Continued monitoring, sustainability planning, and expansion of outcome measurement are recommended to ensure that these process improvements translate into meaningful gains in patient health outcomes.

## Conclusions

This closed-loop clinical audit demonstrated that the implementation of a structured diabetic foot risk assessment and documentation tool, supported by targeted staff education, was associated with significant and consistent improvements in the completeness of diabetic foot assessment, risk stratification, and follow-up documentation at Almanagil Teaching Hospital. The intervention addressed critical gaps in baseline practice, particularly in neuropathy and vascular assessment, infection recognition, objective risk scoring, and care planning, thereby strengthening early identification of patients at high risk of diabetic foot complications. Although patient-level clinical outcomes were not assessed, the marked improvements in standardized assessment and documentation represent important process-of-care gains that are likely to support timely clinical decision-making and improved continuity of care. Sustained audit cycles, ongoing education, and integration of standardized assessment tools into routine practice are recommended to maintain these improvements and to further reduce the burden of preventable diabetic foot complications in similar resource-limited healthcare settings.
